# Bacterial Heavy Metal Resistance in Contaminated Soil

**DOI:** 10.4014/jmb.2411.11073

**Published:** 2025-07-09

**Authors:** Tuyajargal Iimaa, Munkhjin Batmunkh, Batbold Dulguun, Batsuren Dorjsuren, Telmen Turmunkh, Enkhjargal Tserennadmid, Unursaikhan Surenjav, Battsetseg Choidash, Renchinkhand Gereltuya

**Affiliations:** 1 Division of Graduate Education Policy and Management, Mongolian National University of Medical Sciences, Ulaanbaatar 14210, Mongolia; 2 Department of Public Health Reference Laboratory, National Center for Public Health, Ministry of Health, Ulaanbaatar 13381, Mongolia; 3 Laboratory of Air and Environmental Monitoring, Department of Environment and Forest Engineering, School of Engineering and Technology, National University of Mongolia, Ulaanbaatar 14201, Mongolia; 4 Department of Earth and Environmental Sciences, Korea University, Seoul 02841, Republic of Korea; 5 Department of Biology, School of Arts and Sciences, National University of Mongolia, Ulaanbaatar 14201, Mongolia

**Keywords:** Resistance, microorganisms, bioremediation, heavy metals, plasmids

## Abstract

Soil heavy metal contamination poses a significant global threat to both environmental and human health. The accumulation of heavy metals in the Earth's crust, driven by urbanization, industrialization, agricultural practices, improper waste disposal, and mining, triggers harmful ecological cascades. Microorganisms, particularly bacteria, play a vital role in the decomposition and detoxification of these contaminants. In contaminated environments, bacteria exhibit resistance to heavy metals through various strategies, including the production of metal-chelating molecules, alterations to cell surface properties, efflux pumps, and activation of detoxification pathways. Notable microbial species such as *Bacillus*, *Enterobacter*, *Pseudomonas*, *Aspergillus*, and *Penicillium* show promising potential for bioremediation efforts. Harnessing bacterial resistance to heavy metals offers a cost-effective and sustainable approach to mitigate the adverse impacts of contamination. This review explores the mechanisms of heavy metal resistance in bacteria, the role of soil microbiota, and the implications for bioremediation strategies. This review emphasizes the critical importance of bacterial resistance in addressing soil contamination and highlight the need for further research to elucidate underlying mechanisms and enhance bioremediation applications in this urgent global challenge.

## Introduction

Soil is an essential component of the Earth's biosphere, acting as a dynamic ecosystem that sustains a diverse range of organisms, from microorganisms to plants and animals. In recent years, anthropogenic activities—including urbanization, industrialization, agricultural chemical usage, improper waste disposal, mining, and smelting—have contributed to the accumulation of elevated levels of heavy metals in the Earth's crust. This contamination represents a significant and persistent threat to both ecosystems and human health globally [1, 2].

The unique microenvironments within soil offer suitable habitats for diverse microbial populations, each exhibiting distinct adaptive features. The interactions between heavy metals and microorganisms are crucial, as microbial cells can develop mechanisms to mitigate metal stress. For the purposes of this review, "contaminated soil" refers specifically to soils affected by anthropogenic activities leading to elevated heavy metal concentrations beyond natural levels. While many microorganisms capable of heavy metal resistance are found in such environments, similar mechanisms may occur in other habitats, including aquatic and industrial ecosystems. The contamination of soil by heavy metals, pesticides, and organic compounds has reached levels that threaten both human health and environmental integrity. Notably, certain microbial strains—including bacteria, fungi, and algae—have the ability to accumulate heavy metals and contribute to bioremediation efforts [3, 4].

Heavy metal resistance mechanisms are central to this relationship. Microorganisms have evolved sophisticated strategies to survive and thrive in heavy metal-contaminated environments. These mechanisms include metal ion sequestration, efflux systems, enzymatic detoxification, and bioprecipitation, which not only help in neutralizing heavy metal toxicity but also enable microorganisms to play an active role in remediation processes. This interplay underscores the importance of microbial resistance as a cornerstone of bioremediation strategies. This review focuses on bacterial species and their resistance mechanisms in heavy metal-contaminated soils, exploring their critical roles in bioremediation. Bioremediation, which involves the use of microbial processes to remediate contaminated sites, has emerged as an effective and environmentally friendly alternative to conventional chemical and physical remediation techniques. This approach exploits the natural capabilities of microorganisms to degrade or transform contaminants into less harmful substances [5, 6].

The link between bacterial resistance and bioremediation is crucial in addressing heavy metal pollution. Resistant bacteria tolerate and detoxify contaminants, transforming toxic metals into less harmful forms via reduction or methylation. Microbial communities with plasmid-encoded resistance genes enhance remediation by adapting to and neutralizing a broad range of metals.

**Fig. 1:**
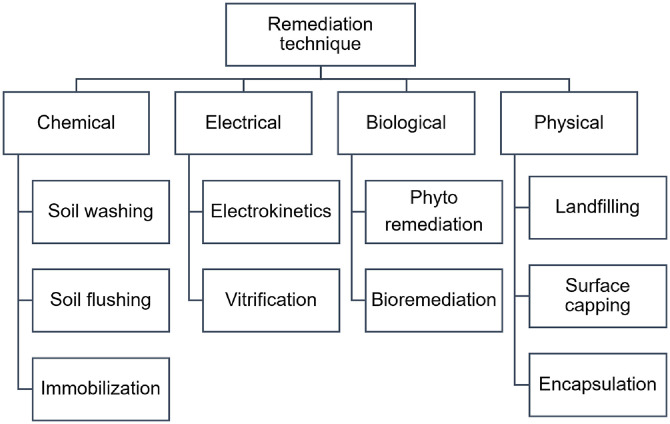
Categories of remediation techniques.

Remediation methods for heavy metal-contaminated sites fall into two categories: ex situ (*e.g.*, landfilling, soil washing, vitrification) and in situ (*e.g.*, phytoremediation, stabilization, electrokinetic extraction). These techniques are further classified into physical, chemical, electrical, and biological approaches (Fig. 1) [7, 8].

In bioremediation, soil microbiota plays a key role, particularly in agriculture. Bacteria reduce or methylate toxic metals, while mycorrhizal fungi assist phytoremediation by promoting plant growth and enhancing metal uptake [9].

However, challenges persist. Incomplete contaminant degradation, secondary contamination, and the need for optimal environmental conditions (*e.g.*, pH, temperature, moisture) limit microbial activity. Mixed contaminants and high pollutant levels can further inhibit growth and reduce effectiveness. Thus, while bioremediation offers a sustainable and cost-effective approach, its success depends on addressing site-specific conditions and limitations [10].

This review provides an overview of heavy metal contamination, bacterial resistance mechanisms, and the roles of soil microbiota and plasmids in bioremediation.

## Heavy Metal Contamination in Soil

The selection of remediation methods for contaminated sites depends on the type, location, and distribution of contaminants [11]. Heavy metals such as cobalt, zinc, chromium, and cadmium accumulate in soil through air, water, and plant cycles, disrupting ecosystems and affecting human health [12]. Unlike air and water, soil has low mobility, leading to slower cross-contamination, yet pollutants enter plant roots, infiltrate the food chain, and persist in the environment. Metals also deposit in soil via precipitation (*e.g.*, rain, snow, fog), with contaminants like mercury, lead, cadmium, and chromium undergoing chemical transformations that influence their toxicity and bioavailability. The mobility and bioavailability of heavy metals in soil are influenced by chemical, physical, and biological processes [13, 14]. In alkaline soils, increased ionization of humus and clay reduces the negative charge of hydroxide ions, iron oxides (Fe_2_O_3_), and aluminium oxides (Al_2_O_3_), enhancing heavy metal absorption [15].

Furthermore, human activities such as industrial emissions, transportation, and the use of phosphate fertilizers contribute to soil contamination [16]. Globally, cadmium levels in agricultural soils increase by approximately 3%annually due to the widespread use of phosphate fertilizers, contributing to long-term soil contamination [17]. Lead contamination in rice grain harvested from smelter-affected area typically ranges 1.35 times higher than the corresponding the maximum permissible level [18].

Recent studies, such as one conducted in Beijing, China, in 2022, analysed the heavy metal content (chromium, nickel, copper, zinc, lead, arsenic, cadmium, and mercury) in street dust from industrial, residential, educational, commercial, and park areas across different seasons. Source apportionment analysis revealed that the heavy metals in the dust primarily originated from mixed sources, including natural, industrial, and traffic-related activities [19]. Similar research conducted in various countries—including Nigeria, Iran, Turkey, Mongolia, and Poland—has documented increased heavy metal contamination due to the accumulation of chemicals and their compounds in solid, liquid, and gaseous forms, influenced by processes such as disintegration, dissolution, absorption, and evaporation in soil [20-24].

Furthermore, numerous studies have documented elevated levels of heavy metal contamination in areas adjacent to roadways. For instance, in Pakistan, heavy metals accumulate in both soil and plant tissues along highways, with lead concentrations in plants ranging from 0.08 to 3.98 mg/kg and in soil from 1.95 to 4.74 mg/kg. Similar patterns of contamination have been observed in other countries, including China, Mongolia, and Australia, where urban areas near roads are particularly susceptible to lead contamination. Generally, a decrease in chemical contamination is noted with increasing distance from roadways. Additionally, the phenomenon of acid rain exacerbates soil acidity, promoting the dissolution of lead and facilitating its interaction with reactive chemical substances, which in turn leads to the formation of soluble complexes [22, 25, 26].

Heavy metal contamination levels vary even within the same urban environment or along different segments of highways, influenced by factors such as traffic density, industrial activity, and local environmental conditions. To ensure comparability across studies, sampling locations are selected based on criteria such as proximity to pollution sources, land use patterns, and prevailing wind directions [27].

## Bacterial Resistance Mechanisms

**Fig. 2:**
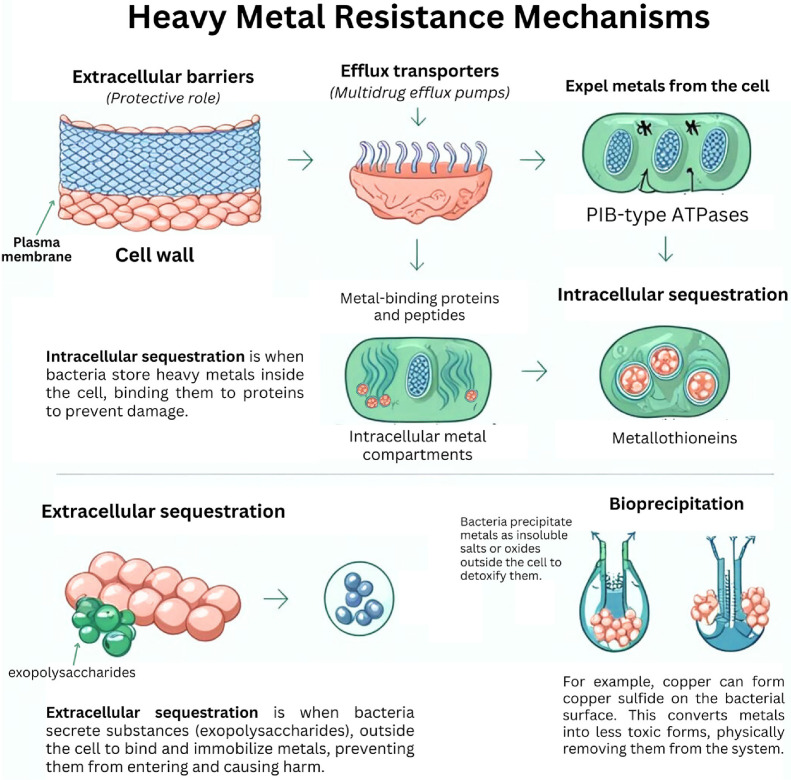
Mechanisms of bacterial heavy metal resistance.

Numerous studies have identified five primary mechanisms by which bacterial strains exhibit resistance to heavy metals. A thorough understanding of these mechanisms is essential for developing effective strategies for the detoxification and removal of heavy metals from contaminated environments (Fig. 2).

In a recent study, EPS from *Streptomyces* sp. MOE6 was shown to reduce the capacity to remove toxic metals such as Co(II), Cr(VI), Cu(II) and U(VI) and from solution either by chelation and/or reduction, highlighting its bioremediation potential [28]. *Bacillus subtilis* has demonstrated resistance to cadmium at concentrations of up to 150 mg/l, while *Pseudomonas aeruginosa* exhibits a higher tolerance, reaching 200 mg/l [29]. Below is a detailed examination of each of the key mechanisms underlying bacterial resistance to heavy metals:

**1. Extracellular barriers:** The impermeability of bacterial cell walls, plasma membranes, and capsules serves as a key resistance mechanism against metal ions. Bacterial resistance to heavy metals begins with physical barriers that prevent metal ions from entering the cell

● Cell wall and plasma membrane: The bacterial cell wall's structure can prevent metal ions from crossing into the cytoplasm. The lipid bilayer of the plasma membrane also contributes to the impermeability against various metal ions.

● Capsule: The outermost layer, the bacterial capsule, often composed of polysaccharides, helps accumulate metal ions. These polysaccharides contain functional groups, such as carbonyl groups, which can coordinate with metal ions, preventing them from entering the cell.

● Protective role: By maintaining this impermeability, bacteria can restrict the influx of potentially toxic metals like cadmium, lead, and copper, which would otherwise interfere with cellular processes. For example, polysaccharides in the capsules of *Pseudomonas*, particularly those with carbonyl groups, effectively accumulate heavy metal ions, providing a protective barrier [30, 31].

**2. Efflux transporters:** Efflux systems are membrane-bound transport proteins that actively expel metal ions from bacterial cells, thereby preventing their accumulation to toxic concentrations. These systems play a crucial role in bacterial resistance to heavy metals, ensuring cellular homeostasis and survival under metal stress conditions.

● PIB-type ATPases: These ATPases are responsible for transporting a variety of metal ions, including copper, zinc, and cadmium, through the bacterial inner membrane. The transport process is driven by ATP hydrolysis, which provides the necessary energy to pump metal ions against concentration gradients. The expression of these transporters is typically upregulated in response to heavy metal exposure, highlighting their role in maintaining metal ion balance within the cell [32].

● Energy-dependent: The efflux process is energy-dependent, relying on ATP to actively transport metal ions out of the cell. This mechanism is essential for preventing the intracellular accumulation of toxic metals that could otherwise interfere with cellular processes and cause damage [33].

● Multidrug efflux pumps: In addition to metal ions, some bacteria possess multidrug efflux pumps capable of exporting a broad range of toxic substances, including antibiotics and heavy metals. This dual-functionality of multidrug efflux pumps confers both antimicrobial and metal resistance. For instance, *Escherichia coli* utilizes the CusA efflux pump for copper resistance, while *Pseudomonas putida* expresses various ATPases to resist zinc and copper [33, 34].

**3. Bioaccumulation and intracellular sequestration:** A key resistance mechanism in bacteria involves the binding of metal ions to intracellular components such as proteins, peptides, and enzymes, which sequester these metals and reduce their toxic effects within the cytoplasm.

● Metallothioneins: Low-molecular-weight proteins enriched with cysteine residues play a crucial role in binding metal ions through thiol (-SH) groups. These metallothioneins are vital for bacterial strains to sequester metals such as zinc, cadmium, and copper. The expression of metallothionein genes is often upregulated in response to heavy metal exposure, helping bacteria maintain metal homeostasis and mitigate toxicity. For example, *Synechococcus elongatus* PCC 7942 and *Anabaena* PCC possess SmtA metallothioneins for zinc resistance, while *Oscillatoria brevis* and *Pseudomonas putida* express BmtA metallothioneins [35, 36].

● Metal-binding proteins and peptides: Other proteins and peptides, such as ferritin and phytochelatins, also bind metal ions, facilitating their safe storage within the bacterial cell. This prevents harmful interactions between metal ions and critical cellular components such as DNA or enzymes. For instance, *P. putida* employs various proteins to sequester copper and cadmium ions [37].

● Intracellular metal compartments: Certain bacteria have evolved mechanisms to compartmentalize toxic metals into storage granules or specialized organelles, further isolating them from essential cellular processes and reducing their potential toxicity [38].

**4. Extracellular sequestration:** Bacteria also resist heavy metals by producing high-molecular-weight exopolysaccharides (EPSs), which are synthesized and secreted outside the cells [33]. EPSs consist of macromolecules—including proteins, polysaccharides, nucleic acids, lipids, and low-molecular-weight compounds—that primarily interact with heavy metal ions through precipitation, tolerance, conjugation, and self-protection mechanisms. This external mechanism involves biosorption, precipitation, and conjugation [39, 40].

● Exopolysaccharides (EPSs): These high-molecular-weight macromolecules are secreted into the surrounding environment, where they can bind to metal ions through precipitation or other forms of complexation. This reduces the bioavailability of metals near the bacterial surface [41, 42].

● Biosorption: This process involves the adsorption of metal ions onto the bacterial surface. It can occur through ion exchange, diffusion, and adsorption mechanisms, with different bacteria using various functional groups (*e.g.*, carboxyl or phosphate groups) to bind the metal ions. Gram-positive bacteria typically utilize carboxyl groups of peptidoglycans for biosorption, while gram-negative bacteria rely on phosphate groups [43].

● Exopolysaccharides and precipitation: In some cases, EPSs form insoluble metal complexes, making them less toxic to the cell. For instance, *Pseudomonas syringae* produces copper-binding proteins like CopA and CopB, resulting in the visible blue coloration of colonies when copper ions are accumulated. *Bacillus* species often release EPSs that bind cadmium and lead [44].

**5. Bioprecipitation:** Bacteria can facilitate the formation of insoluble metal compounds, effectively reducing the bioavailability and toxicity of heavy metals in the environment. This process, known as bioprecipitation, involves the generation of solid metal compounds that can be removed from contaminated environments.

● Precipitation of metal salts: Bacteria can induce the precipitation of heavy metals by altering local pH or through biochemical reactions that lead to the formation of solid metal compounds, such as phosphate salts. This process effectively immobilizes toxic metals, preventing them from entering the food chain or leaching into water sources.

● Role of phosphates and sulfides: Certain bacterial species generate phosphate or sulfate ions that interact with metal ions to form insoluble salts. For instance, *Staphylococcus aureus* can precipitate lead as lead phosphate (Pb_3_(PO_4_)_2_), while *Vibrio harveyi* also precipitates lead as lead phosphate (Pb_9_(PO_9_)_6_). Similarly, *Desulfovibrio desulfuricans* can precipitate mercury as mercury sulfide (HgS). Precipitation of metals like cadmium and mercury, via sulfide and phosphate interactions, represents a vital mechanism in environmental detoxification [45].

● Environmental detoxification: Bioprecipitation plays an essential role in detoxifying environments contaminated with heavy metals such as lead, cadmium, and mercury. For example, *S. aureus* can precipitate lead as lead phosphate (Pb_3_(PO_4_)_2_), and *V. harveyi* can precipitate lead as lead phosphate (Pb_9_(PO_9_)_6_). *D. desulfuricans* can precipitate mercury as mercury sulfide (HgS). These findings highlight bioprecipitation's potential for minimizing lead contamination and restoring affected environments [45-47].

Research by Bender *et al*. (2004) indicates that bacterial consortia employing multiple resistance mechanisms enhance bioremediation efficiency. For instance, *Pseudomonas*, *Bacillus*, and *Acinetobacter* consortia effectively detoxify cadmium and lead through biosorption, bioaccumulation, and bioprecipitation. Similarly, *Rhodococcus*, *Sphingomonas*, and *Microbacterium* species degrade organic contaminants while transforming heavy metals in soil [5]. Additionally, the combination of arbuscular mycorrhizal fungi (AMF) with *Pseudomonas fluorescens* has been shown to enhance heavy metal uptake in plants through phytoremediation, promoting both plant growth and soil health [48]. Further exploration of these bacterial resistance mechanisms will aid in developing advanced strategies for mitigating heavy metal contamination (Table 1).

## Role of the Soil Microflora in Ecosystem Functioning

Soil, as the foundation of terrestrial ecosystems, is a dynamic environment that sustains a diverse range of microorganisms. These microorganisms perform critical ecological functions that encompass nutrient transformation, organic matter decomposition, pollutant degradation, and plant growth promotion, among other activities [49, 50].

**Nutrient cycling.** Soil microorganisms are pivotal in nutrient cycling processes, transforming organic and inorganic materials into bioavailable forms.

● **Bacteria.** Soil bacteria play essential roles in biogeochemical cycles, thriving on diverse nutrient sources within various microenvironments. Common soil bacteria include *Acinetobacter*, *Agrobacterium*, *Bacillus*, *Clostridium*, *Pseudomonas*, and *Xanthomonas*, among others. Nitrogen-fixing bacteria (*e.g.*, *Rhizobium* and *Azotobacter*) convert atmospheric nitrogen into ammonia, which plants can assimilate. Nitrifying bacteria (*e.g.*, *Nitrosomonas* and *Nitrobacter*) oxidize ammonia to nitrates, while denitrifying bacteria (*e.g.*, *Pseudomonas* and *Paracoccus*) return nitrogen to the atmosphere as nitrogen gas. Sulfur-reducing bacteria (*e.g.*, *Desulfovibrio*) and iron-reducing bacteria (*e.g.*, *Geobacter*) also contribute to critical redox processes in soil [51, 52]. Metal-resistant bacteria, such as *Cupriavidus metallidurans* and *Ralstonia eutropha*, are key players in the detoxification of contaminated environments. These bacterial consortia possess specialized mechanisms that enable them to survive and thrive in environments with high concentrations of heavy metals, thereby facilitating the transformation and immobilization of toxic metals into less harmful forms. The interactions among these microbial species contribute to the synergistic breakdown of metal contaminants, enhancing their removal and recovery, which is essential for effective bioremediation. The functional diversity of these microbial communities allows for the degradation, stabilization, or sequestration of heavy metals, making them vital in reducing the environmental and health risks posed by metal contamination [7, 28].

● **Actinomycetes.** These microorganisms, particularly *Streptomyces*, decompose complex organic compounds like cellulose and lignin, releasing nutrients such as nitrogen, phosphorus, and sulfur into the soil. Key genera include *Streptomyces*, known for their filamentous structures resembling fungi, and *Mycobacterium*, characterized by rod-shaped and cocci-shaped cells [53-55].

● **Molds.** Molds thrive in well-ventilated, acidic environments and can exist independently or form mycorrhizal associations with plant roots. They include species from the genera *Aspergillus*, *Geotrichum*, *Penicillium*, and *Trichoderma*, as well as various ascomycetes and basidiomycetes [56]. Molds in soil exhibit active metabolism under favorable moisture, temperature, and nutrient conditions, with many relying on carbohydrates while some are capable of decomposing plant residues and lignin. Under adverse conditions, molds can enter a dormant state, surviving for decades as spores [57, 58].

● **Protozoa.** Soil protozoa, including amoebae, flagellates, ciliates, and heliozoa, are diverse unicellular eukaryotic organisms inhabiting soil environments. They act as bioindicators for soil quality and play a role in nutrient cycling by preying on bacteria and other microorganisms. This grazing activity facilitates nutrient turnover, particularly for nitrogen and phosphorus, making these nutrients more accessible to plants. Common examples include amoebae such as *Acanthamoeba* and *Hartmannella*, ciliates like *Paramecium*, and flagellates like *Cercomonas* [58, 59]. Soil protozoa interact with microbial communities through predator-prey dynamics, shaping microbial diversity and ecosystem functioning.

● **Algae.** *Cyanobacteria* enhance soil fertility through nitrogen fixation, while green algae and diatoms contribute to carbon cycling by sequestering carbon during photosynthesis. Research highlights that soil algae account for up to 30% of carbon fixed through photosynthesis in certain ecosystems [60]. *Cyanobacteria*, in particular, enhance soil fertility through nitrogen fixation, while some algae demonstrate potential as biofuel sources due to their high lipid content [61].

**Bioremediation of pollutants.** Metal-resistant bacteria such as *Pseudomonas* and *Bacillus* detoxify heavy metals through reduction, methylation, and chelation. Hydrocarbon-degrading bacteria break down oil spills and other organic pollutants [62, 63]. While specific field studies involving both *P. putida* and *B. subtilis* used together for the remediation of cadmium and lead-contaminated soils are limited, several studies highlight the effectiveness of these bacteria individually in heavy metal bioremediation. For instance, the study examined the removal of lead, cadmium, and zinc from contaminated agricultural soil by *B. subtilis*. The study reported removal efficiencies ranging from approximately 75.7% to 78.25% for lead over a period of 14 to 35 days, and 59.64% for cadmium at 35 days. This indicates that *B. subtilis* can be effective in remediating lead and, to a lesser extent, cadmium in contaminated soils. Furthermore, a study investigated the bioaugmentation potential of toxic metal-tolerant bacteria isolated from contaminated soils [64]. The researchers identified *Pseudomonas aeruginosa* and *B. cereus* as highly tolerant to cadmium and lead. Pot experiments demonstrated that inoculation with *P. aeruginosa* strains significantly improved the growth of *Oryza sativa* (rice) seedlings in cadmium- and lead-contaminated soils, suggesting the bacterium's potential in mitigating heavy metal toxicity in agricultural settings [65]. While these studies focus on the individual application of metal-resistant bacterial strains, they collectively highlight the potential of using such bacteria in bioremediation strategies to reduce heavy metal concentrations in contaminated environments.

Certain molds degrade persistent organic pollutants, such as pesticides and hydrocarbons, into less toxic forms [57, 58].

**Adaptation of environmental extremes.** Soil microorganisms exhibit remarkable resilience and adaptability to varying environmental conditions, maintaining ecosystem functionality. Psychrophiles as cold-tolerant species thrive in polar and alpine soils, contributing to nutrient cycling under extreme conditions [66]. Drought-resistant species exhibit adaptations to arid environments, maintaining soil health during prolonged dry periods [67].

**Organic matter decomposition.** Microorganisms are the primary agents of organic matter decomposition, breaking down plant residues and animal waste into simpler compounds. Molds such as *Aspergillus* and *Penicillium* decompose lignin and other recalcitrant organic materials, facilitating nutrient release and soil organic matter formation [56]. *Bacillus* species and actinomycetes degrade complex organic molecules, promoting humus formation and enhancing soil fertility [51, 53].

**Soil structure improvement.** Fungi hyphae produce extracellular polysaccharides that bind soil particles, forming stable aggregates. Bacteria secrete biofilms and other adhesive substances, which enhance soil aggregation and water retention [68, 69].

**Plant growth promotion.** Some microorganisms enhance plant growth by facilitating nutrient uptake, producing growth-promoting hormones, and suppressing plant pathogens. Plant growth-promoting rhizobacteria (PGPR), including species of *Pseudomonas* and *Bacillus*, produce auxins, gibberellins, and siderophores, improving plant nutrient acquisition. Mycorrhizal fungi form symbiotic associations with plant roots, extending the root system’s reach and improving water and nutrient uptake [68].

**Disease suppression.** Microbial communities in soil naturally suppress plant pathogens through competition, production of antimicrobial compounds, and induction of plant resistance. Antagonistic bacteria such as *Pseudomonas fluorescens* produce antibiotics that inhibit pathogenic fungi and bacteria. Biocontrol fungi, such as *Trichoderma*, parasitize plant pathogens and secrete enzymes that degrade their cell walls [68, 69].

## Bacterial Species Resistant and Their Bioremediation Applications

A total of 59 strains of actinobacteria were isolated from a mining area in Morocco, and their resistance to lead, zinc, cadmium, copper, and chromium was assessed using microbiological assays (morphological, physiological, and biochemical characterizations) and instrumental methods, such as atomic absorption spectrometry and the spread plate method. Notably, *Streptomyces* sp. BN2 demonstrated resilience to lead concentrations up to 0.55 mg/kg and hexavalent chromium concentrations of up to 0.1 mg/kg [70]. In Brazil, species such as *Gemella* sp. and *Micrococcus* sp. were identified as resistant to lead and cadmium, while *Hafnia* sp. exhibited notable resistance to cadmium [71].

Some studies have shown that heavy metal ions preferentially bind to functional groups in the bacterial cell wall, including amino, carboxyl, hydroxyl, phosphate, sulfate, and amine groups. The primary resistance mechanism involves the adsorption of metal ions to these reactive groups, facilitating their sequestration within the cell protoplast. Gram-positive bacteria generally exhibit higher metal accumulation due to the presence of glycoproteins in their cell walls, whereas Gram-negative bacteria show comparatively lower absorption due to the structural components of their cell walls, specifically phospholipids and lipopolysaccharides [72, 73].

Microorganisms employ various strategies to tolerate heavy metal contamination, including genetic adaptations and structural modifications. They interact with heavy metals through biochemical processes such as oxidation, reduction, alkalization, and dealkalinization. The following section summarizes bacterial resistance to several chemicals:

**As-resistant bacteria.** Arsenic contamination presents a significant environmental challenge. Studies in Indonesia and Malaysia have identified several arsenic-resistant bacterial species, including *Enterobacter* sp.(MNZ1), *Klebsiella pneumoniae* (MNZ4 and MNZ6), *Arthrobacter globiformis*, *Enterobacter asburiae*, *Aeromonas* sp., *Acinetobacter* sp., *Pseudomonas* sp., *Bacillus megaterium*, *Bacillus cereus*, *Bacillus pumilus*, *Staphylococcus lentus*, *Exiguobacterium* sp., and *Escherichia coli*. Notably, *Rhizobium radiobacter*, often associated with plant roots, demonstrated a minimum inhibitory concentration exceeding 1500 mg/kg for arsenic [74, 75].

**Hg-resistant bacteria.** Microorganisms have evolved multiple detoxification strategies for mercury-contaminated environments, primarily involving reduction and volatilization processes. In Indonesia, *Brevundimonas* sp. strains HgP1 and HgP2, isolated from gold mining sites, exhibited significant mercury resistance. Research by Parisa Keramati *et al*. identified bacterial genera including *Staphylococcus*, *Bacillus*, *Pseudomonas*, *Citrobacter*, *Klebsiella*, and *Rhodococcus* as effective for restoring mercury-degraded ecosystems [76, 77].

**Cd-resistant bacteria.** Cadmium-resistant bacteria, such as *Salmonella enterica* (strain 43C) and *R. metallidurans* (strain CH34), have been identified in contaminated environments. The CH34 strain effectively reduced soil cadmium levels. Other resistant strains include *Pseudomonas aeruginosa* and *Staphylococcus xylosus* [78, 79]. Research has also focused on using *Bacillus thuringiensis* to reduce cadmium and lead contamination in agricultural field.

**Pb-resistant bacteria.** Lead-resistant bacteria such as *Bacillus* sp., *Pseudomonas* sp., *Corynebacterium* sp., and *R. metallidurans* have demonstrated the ability to reduce lead toxicity. *B. subtilis* (strain X3) tolerated lead salt concentrations up to 2,000 mg/l, while *R. metallidurans* (CH34) reduced soil lead concentration from 3.5 mg/l to 0.2 mg/l. Additional strains, including *Pseudomonas aeruginosa* and *Gemella* sp., have shown resistance at various concentrations [71, 80].

**Ni-resistant bacteria.** In Iran, *Cupriavidus* sp., isolated from industrial wastewater, demonstrated significant nickel resistance, confirmed through morphological, biochemical, and 16S rDNA gene sequencing analyses. Other resistant strains included *Klebsiella oxytoca* *ATHA6*, which tolerated nickel concentrations up to 83 mg/ml [81, 82].

**Cu-resistant bacteria.** Strains of *Sphingomonas*, *Stenotrophomonas*, and *Arthrobacter* isolated from copper-contaminated agricultural soils in Chile showed resistance to copper salts. *Bacillus pumilus* exhibited resistance at a concentration of 121.82 mg/l, and *Staphylococcus pasteuri* at 80 mg/l. These bacteria demonstrate mechanisms that enable them to tolerate and potentially remediate heavy metal contamination [83, 84]. A comprehensive summary of the identified heavy metal-resistant bacterial species is presented in Table 2.

## Resistance MIC of Bacterial Strains

The minimum inhibitory concentration (MIC) is an important parameter in the evaluation of bacterial heavy metal tolerance. Most resistant bacterial genera against arsenic, cadmium, and lead were *Pseudomonas*, *Agrobacterium*, *Bacillus*, *Klebsiella*, *Enterobacter*, *Microbacterium*, and *Rhodococcus*. Among these, *Enterobacter aerogenes* K6 and *K. pneumoniae* K5 exhibited the highest resistance to cadmium and lead, and *Agrobacterium* and *Bacillus* showed highest resistance to arsenite [85]. Quantitative studies highlight significant variability in bacterial resistance MIC across genus is presented in Table 3.

## Bioremediation Efficiencies

The adsorption of metal ions was significantly affected by the initial pH of solution, initial metal ion and polysaccharide concentrations, and presence of other ions in solution. At optimum pH, the uptakes of Pb, Cu and Zn on the polysaccharide producing *Bacillus firmus* were 98.3%, 74.9% and 61.8%, respectively. The metal ions removal was lower at neutral and generally the initial adsorption rate was rapid and reached equilibrium after 10 min [86]. Also at optimum pH, the uptakes of the metals on the polysaccharide producing *Azotobacter chroococcum* were 40.48% and 47.87% respectively [87]. The metal ions biosorption was high at 4, 5-5. *Ensifer meliloti* MS-125 adsorbed 89, 85 and 66 % of lead, nickel and zinc, respectively [88] . Quantitative studies of absorption of metal ions by EPS producing bacteria were presented in Table 4. Based on a comparative analysis (Table 5), *Alcaligenes faecalis* exhibits superior bioremediation potential compared to *Bacillus cereus*, primarily due to its higher metal uptake efficiency, enhanced tolerance to chromium, and more efficient enzymatic detoxification mechanisms [5, 7, 89, 90].

## R-Plasmids

Bacterial plasmids, known as R-plasmids, are extrachromosomal genetic elements that play a crucial role in conferring heavy metal tolerance. While many heavy metal resistance genes are plasmid-mediated, not all mechanisms are. For instance, *Pseudomonas* sp. exhibited resistance to lead at concentrations up to 1,000 mg/ml, but no plasmids conferring tolerance were detected, suggesting chromosomal genes or alternative mechanisms may be involved [91, 92].

Heavy metal resistance genes often co-localize with antibiotic resistance genes on plasmids, facilitating the horizontal transfer of resistance traits among bacteria. These plasmids mediate resistance through various mechanisms, such as efflux pumping, redox chemistry, metal exclusion, and activation of resistance pathways. For instance, *S. aureus* contains a plasmid-encoded cad system that effluxes cadmium ions, providing resistance [91, 93, 94].

Since 1991, numerous studies have established a correlation between heavy metal resistance and antibiotic resistance across various bacterial species. Environments contaminated with heavy metals frequently harbour elevated levels of antibiotic-resistant bacteria. This co-resistance highlights the dual adaptive advantages conferred by plasmids, enabling bacterial survival in complex environments with mixed contaminants [95-97].

Plasmid-mediated traits are particularly significant in bioremediation systems, where microbial adaptability is essential for efficient remediation of environments contaminated with both heavy metals and antibiotics. These plasmids can enhance microbial survival and metabolic versatility, enabling bacteria to detoxify and metabolize diverse contaminants simultaneously. For example, bacteria harbouring plasmids with both heavy metal resistance and antibiotic resistance genes can thrive in polluted sites, such as industrial effluents and agricultural soils. Such traits are critical for the stabilization and reclamation of these ecosystems [98-100] Moreover, genetic elements on plasmids, such as transposons and integrous, facilitate the acquisition and dissemination of resistance genes within microbial communities. This genetic flexibility underpins the resilience of microbial consortia in bioremediation processes. Understanding the role of R-plasmids in mediating microbial adaptability provides valuable insights into engineering bacterial strains or consortia for targeted bioremediation applications [101-103]. The study details a mutant *Escherichia coli* strain (8mM-CRAA) with a minimum inhibitory concentration of 8 mM cadmium was generated, which was approximately eightfold higher than the minimum inhibitory concentration of the wild-type *E. coli* BL21(DE3) strain [104]. Researchers discuss *C. metallidurans* CH34 contains 12 predicted RND proteins, which are part of transenvelope protein complexes that export toxic metal cations from the periplasm to the outside, contributing to metal resistance. Genome analysis revealed that *C. metallidurans* CH34 has significantly more putative transition metal transport systems than six related proteobacteria, with these systems located on its chromosome 2 and plasmids pMOL28 and pMOL30. The resistance determinants for cobalt, nickel, chromate, and zinc on plasmid pMOL28, and for copper, lead, and mercury on plasmid pMOL30, evolved through gene duplication and horizontal gene transfer events, which enhanced the strain's adaptation to serpentine-like soils [105].

Table 6 presents examples of specific bacterial species with identified heavy metal resistance plasmids, underscoring the remarkable adaptability of microorganisms to diverse environmental stressors. These findings offer promising avenues for optimizing bioremediation strategies, particularly in environments with mixed contaminants.

## Limitations and Future Perspectives

Despite the significant progress in understanding bacterial resistance mechanisms and their applications in bioremediation, several challenges and knowledge gaps remain. Addressing these issues is essential for enhancing the efficiency and scalability of bioremediation strategies.

Diversity of resistance mechanisms. While mechanisms such as efflux pumps, bioaccumulation, and bioprecipitation are well-characterized, the molecular pathways in less-studied bacterial species remain unexplored. This limits the discovery of novel mechanisms that could enhance bioremediation efficiency [106, 107].

Microbial community dynamics. The interactions within microbial consortia in contaminated soils are poorly understood. Synergistic relationships between species such as *Pseudomonas aeruginosa* and *B. subtilis* could unlock more effective strategies for remediation.

Adaptation to mixed contaminants. Heavy metals often co-occur with other pollutants like hydrocarbons and antibiotics. Research on bacterial co-resistance and adaptability to such complex environments is still in its infancy [96].

Scalability issues. Laboratory studies rarely replicate the complexity of field conditions. Soil heterogeneity, climate variability, and pollutant distribution significantly affect bioremediation outcomes. For instance, while *Pseudomonas* strains are effective in controlled environments, their efficiency under field conditions is inconsistent [108].

Environmental and ecological constraints. Introducing resistant bacterial strains into ecosystems could disrupt native microbial communities, leading to unforeseen ecological consequences. Additionally, the risk of horizontal gene transfer of resistance traits raises biosecurity concerns [109]. Some studies emphasize the complexities and challenges inherent in bioremediation projects. A study investigated the bioaugmentation of oil-polluted soils from Kuwait, Lebanon, Egypt, and Germany using indigenous hydrocarbon-degrading microorganisms from Kuwaiti environments. The results indicated that bioaugmentation did not enhance oil removal in soils from Kuwait, Egypt, and Germany. The failure was attributed to the inability of the introduced microbial strains to adapt and compete with the native microbial communities, highlighting the challenge of microbial competition and adaptation in bioaugmentation strategies [110]. Another study attempted to enhance nitrogen removal in a sequencing batch reactor by inoculating it with the aerobic denitrifying bacterium *Microvirgula aerodenitrificans*. Despite two inoculations, no improvement was observed. The introduced bacteria were rapidly eliminated, primarily due to predation by protozoa, underscoring the importance of understanding microbial interactions and predator-prey dynamics in bioremediation efforts [111].

Economic constraints. Large-scale microbial bioremediation requires substantial investments in microbial cultivation, monitoring, and maintenance, posing challenges for widespread adoption in low-resource settings [112].

## Conclusion

Heavy metal contamination, primarily resulting from anthropogenic activities, presents a significant threat to human, animal, and plant health. These metals accumulate in the Earth's surface layer, leading to profound alterations in soil composition and functionality. However, considerable progress has been made in identifying various microorganisms, including bacteria, fungi, and algae, that exhibit the potential for heavy metal tolerance and the development of resistance mechanisms. Many of these microorganisms possess the ability to neutralize and detoxify heavy metal contaminants effectively.

Biological remediation, which employs microorganisms to degrade and remediate contaminated sites, is emerging as a promising and environmentally sustainable strategy for mitigating heavy metal pollution. This application of microbial agents can significantly reduce contaminant levels in polluted soils to safe and acceptable thresholds. Key conclusions from this research are as follows: (1) Microorganisms such as bacteria and fungi play a pivotal role in neutralizing and detoxifying heavy metals, offering a sustainable approach to mitigate contamination. (2) Biological remediation is a promising strategy for reducing heavy metal pollution, highlighting its importance for environmental restoration. (3) Diverse mechanisms, including efflux systems, bioprecipitation, and extracellular barriers, are utilized by bacteria to counteract heavy metal toxicity, showcasing their adaptability and utility in bioremediation.

For challenges in bioremediation: (1) The effectiveness of microbial bioremediation in field conditions remains inconsistent due to environmental variability and complex contaminant interactions. (2) Limited scalability of laboratory-based findings to real-world applications poses a significant hurdle. (3) Mixed contaminations, such as co-occurrence of heavy metals and antibiotics, demand a deeper understanding of microbial co-resistance mechanisms.

For future research directions: (1) Development of field-scale bioremediation systems that integrate microbial consortia with tailored resistance capabilities. (2) Exploration of genetic engineering and synthetic biology to enhance microbial efficiency and adaptability. (3) Long-term monitoring and assessment of bioremediation processes to optimize their effectiveness and sustainability. (4) Investigation of the interplay between heavy metal resistance and other environmental stressors to develop holistic remediation strategies.

By addressing these challenges and leveraging microbial resistance mechanisms, future advancements in bioremediation can achieve more effective restoration of contaminated soils and safeguard ecosystems. This progress will ensure human health is protected from the detrimental effects of heavy metal contamination, paving the way for sustainable environmental management. In summary, advancing our understanding of microbial remediation mechanisms, coupled with technological innovations, paves the way for a future in which contaminated soils are restored, ecosystems flourish, and human health is protected from the harmful effects of heavy metal contamination.

## Figures and Tables

**Table 1:** Comparative summary and environmental considerations.

Mechanism	Advantages	Best for	Environmental conditions
Metal binding by cell wall	Non-energy dependent, broad spectrum	Pb^2+^, Cd^2+^, Cr^3+^	Moderate to high metal concentrations
Efflux pumps (ATPases)	Energy-dependent, selective for specific metals	Cu^2+^, Zn^2+^, Cd^2+^	High metal contamination, requires energy
Reduction/Oxidation	Converts toxic metals to less toxic forms	Cr^6+^ → Cr^3+^, Hg^2+^ → Hg^0^, As^5+^ → As^3+^	Anaerobic, low-oxygen, high organic matter
Biosorption by EPSs	Non-toxic, large surface area, efficient for multiple metals	Pb^2+^, Cd^2+^, Cu^2+^	Moderate to low metal concentrations, biofilms

**Table 2:** Summary of heavy metal-resistant bacterial species.

Heavy metal resistance	Bacteria species	References
Lead	*Arthrobacter* sp, *Stapylococcus* sp, *Bacillus subtilis* X3, *Pseudomonas aeruginosa*, *Bacillus cereus*, *Pseudomonas putida*, *Alcaligenes eutrophus*, *Trichoderma* sp and *Stenotrophomonas maltophilia*	[70, 71, 80, 82, 93]
Cadmium	*Pseudomonas aeruginosa*, *Cupriavidus* sp.strain, *Synechococcus* sp, *Pseudomonas veronii*, *Desulfovibrio desulfurican*, *Arthrobacter* sp, *Trichoderma* sp and *Escherichia coli*	[71, 78-84, 91]
Nickel	*Pseudomonas aeruginosa*, *Bacillus* sp. KL1, *Cupriavidus* sp. ATHA3, *Klebsiellaoxytoca* ATHA6, *Metylobacterium* sp. and *Methylobacterium* sp ATHA7	[81, 82, 97]
Zinc	*Pseudomonas aeruginosa*, *Desulfovibrio desulfuricans*, *Pseudomonas aeruginosa*, *Synechococcus* sp and *Escherichia coli*	[97]
Mercury	*Escherichia coli*, *Alcaligenes faecalis*, *Bacillus pumilus*, *Pseudomonas aeruginosa*, and *Brevibacterium iodinium*, *Bacillus firmus*, *Bacillus* sp, *Lysinibacillus fusiformis* and *Serratia marcescens*	[76, 77]
Copper	*Pseudomonas putida*, *Desulfovibrio desulfuricans*, *Sphingomonas*, *Stenotrophomonas*, *Arthrobacter* sp, *Cupriavidus metallidurans*, *Trichoderma* sp and *Salmonella enterica*	[40, 83, 84]
Arsenic	*Enterobacter* sp. (MNZ1), *K. pneumoniae* 1 (MNZ4) and *Klebsiella pneumonia* 2 (MNZ6), *Arthrobacter globiformis*, *Bacillus megaterium*, *Bacillus cereus*, *B. pumilus*, *Staphylococcus lentus*, *Enterobacter asburiae*, *Cupriavidus necator* and *Rhizobium*. *R. Radiobacter*	[74, 75, 95]

**Table 3:** Quantitative studies highlight significant variability in bacterial resistance MIC across genus.

Bacterial genus	Heavy metal	Resistance MIC (mM)	Reference
*Enterobacter aerogenes* K6	Cadmium (Cd)	36	[85]
*Enterobacter aerogenes* K6	Lead (Pb)	18	
*Klebsiella pneumoniae* K5	Cadmiun (Cd)	36	
*Burkholderia*	Lead (Pb)	3.86	
Agrobacterium and Bacillus	Arsenic (As)	45	

**Table 4:** Comparative data showing heavy metal removal efficiencies of soil bacteria.

Bacterial species	Heavy metals adsorbed	Remediation efficiency	Reference
*Bacillus firmus*	Lead, Copper, Zinc	1103 mg Pb^2+^/g EPS (98.3%) 860 mg Cu^2+^/g EPS (74.9%) 722 mg Zn^2+^/g EPS (61.8%)	[86]
*Azotobacter chroococcum*	Lead, Mercury	40.48% Pb^2+^ (33.5 mg/g EPS) 47.87% Hg^2+^ (38.9 mg/g EPS)	[87]
*Ensifer meliloti*	Lead, Nickel, Zinc	89% Pb^2+^ 85% Ni^2+^ 66% Zn^2+^ reduction from 50 ppm initial load	[88]

**Table 5:** Comparative Analysis of *Bacillus cereus* and *Alcaligenes faecalis* in Chromium Bioremediation.

Aspect	*Bacillus cereus*	*Alcaligenes faecalis*
Metal uptake efficiency	Moderate uptake efficiency using surface binding sites.	Higher uptake efficiency due to specialized transport systems.
Metal tolerance levels	Moderate tolerance with efflux pumps and metal-binding proteins. Generally outperformed by other bacterial strains when chromium concentrations are high.	Superior tolerance, with chromate reductase activity that reduces Cr (VI) to Cr (III). The higher tolerance, to thrive in Cr-contaminated environments where other species might fail.
Enzymatic detoxification pathways	Chromium reductase enzymes reduce Cr (VI) to Cr (III) but at a slower rate.	Higher levels of chromate reductase and siderophore production for enhanced sequestration. To produce siderophores, which further enhance chromium sequestration by binding to the metal and reducing its bioavailability.
Bioremediatio n efficiency	Less effective in environments with high chromium concentrations.	More effective in chromium-contaminated environments, demonstrating superior bioremediation potential.

**Table 6:** Heavy metal resistance plasmids.

Bacterial species	Plasmid name	Heavy metals name and decreasing percent, after 90 days treatment				Reference
*Bacillus subtilis*	KSB7	Pb, 53.42%	Zn, 58.46%	Cu, 83.15%	Cr, 84.94%	[113]
